# Efficacy of Postoperative Oral Nutritional Supplements in Geriatric Hip Fracture Patients Undergoing Total Hip Arthroplasty

**DOI:** 10.3390/jcm13185580

**Published:** 2024-09-20

**Authors:** Chang-Hyun Kim, Byung-Ryul Lee, Jong-Seok Park, Jun-Bum Kim, Sai-Won Kwon, Woo-Jong Kim, Ki-Jin Jung, Byung-Woong Jang, Chang-Hwa Hong

**Affiliations:** 1 Department of Orthopaedic Surgery, Soonchunhyang University Hospital Cheonan, 31, Suncheonhyang 6-gil, Dongnam-gu, Cheonan 31151, Republic of Korea; osdrchkim@gmail.com (C.-H.K.); 129027@schmc.ac.kr (B.-R.L.); jsparksch@schmc.ac.kr (J.-S.P.); kjbos@schmc.ac.kr (J.-B.K.); osos@schmc.ac.kr (S.-W.K.); kwj9383@hanmail.net (W.-J.K.); overmas99@hanmail.net (K.-J.J.); 2 Department of Orthopaedic Surgery, Soonchunhyang University Hospital Seoul, 31, Daesagwan-ro 31-gil, Yongsan-gu, Seoul 04401, Republic of Korea; 99845@schmc.ac.kr

**Keywords:** geriatrics, hip fractures, oral nutritional supplements, nutritional status, postoperative complications

## Abstract

**Background/Objectives**: Geriatric hip fracture patients have an increased risk of malnutrition. Proper nutritional supply in the perioperative period is very important for their recovery. Oral nutritional supplements (ONSs) are recommended in geriatric hip fracture patients to improve dietary intake and prevent complications. This study aimed to evaluate the efficacy of postoperative ONSs after total hip arthroplasty in geriatric hip fracture patients. **Methods**: A retrospective study of elderly patients who underwent total hip arthroplasty for hip fracture was conducted. Data from patients who received ONSs postoperatively until hospital discharge (ONS group, n = 69) were compared with patients who did not receive ONSs (control group, n = 168). Laboratory test results, including serum protein and albumin levels, length of hospital stay, and the incidence of postoperative medical complications of the two groups, were analyzed. **Results**: Preoperative serum protein and albumin levels were significantly higher in the control group (*p* = 0.002 and *p* = 0.010, respectively). However, the degree of decline for both protein and albumin levels was significantly less in the ONS group (*p* < 0.001 for both). Serum albumin levels were significantly higher in the ONS group at postoperative two-week follow-up (*p* = 0.006). The length of hospital stay was shorter in the ONS group (*p* < 0.001). The incidence of postoperative delirium was significantly higher in the control group (*p* = 0.007). **Conclusions**: In geriatric hip fracture patients, postoperative ONSs can improve postoperative nutritional status, shorten the length of hospital stay, and reduce the incidence of postoperative delirium.

## 1. Introduction

Geriatric patients undergoing orthopedic surgery are at a high risk of malnutrition due to inadequate dietary intake and insufficient protein supply. In particular, more than half of hip fracture patients are nutritionally deficient [1]. Catabolic effects caused by acute trauma, immobilization, and underlying disease can further worsen their nutritional status. Malnutrition in these patients could delay recovery from illness and increase mortality and morbidity [2]. Malnutrition can also be a major contributing factor to the development of postoperative sarcopenia and frailty, so it can be the most detrimental comorbid condition in geriatric patients. Therefore, nutritional support in geriatric hip fracture patients is crucial to improving their postoperative recovery and preventing complications.

Oral nutritional supplements (ONSs) are products designed to provide a variety of nutrients to individuals whose dietary intake is insufficient to meet daily nutritional requirements. ONSs are recommended for geriatric hip fracture patients to improve dietary intake and prevent complications [3]. ONSs are not only beneficial for patients with malnutrition but can also help prevent the occurrence of nutritional deficiency among patients with reduced postoperative dietary intake [4]. Some studies have found beneficial effects of ONSs in geriatric hip fracture patients on clinical outcome, length of hospital stay, postoperative complications, and even mortality [5,6,7]. In various healthcare settings, ONSs have the advantage of having good compliance and improving the nutritional status regardless of the administration duration [8]. Despite these advantages, studies on the efficacy of ONSs remain mixed, and a 2016 Cochrane review concluded that the evidence of the effectiveness of ONSs after surgery for hip fracture is little [9]. Furthermore, nutritional care was not a priority in the management of geriatric patients with hip fractures. Therefore, further research on the positive effects of postoperative ONSs in these patients is needed. This study aimed to evaluate whether postoperative administration of ONSs could be beneficial to geriatric hip fracture patients.

## 2. Materials and Methods

This retrospective study evaluated the efficacy of ONS administration after total hip arthroplasty (THA) in geriatric hip fracture patients. Medical records of a total of 357 patients who were admitted to our hospital for the diagnosis of hip fracture and undergoing THA surgery from July 2020 to June 2023 were reviewed. Patients under 60 years of age, those with multiple fractures, periprosthetic fractures, ASA classification 4 or higher, postoperative ICU admission, or patients who were transferred for other medical treatment during hospitalization were excluded. Patients who received parenteral nutrition during hospitalization due to an inability to maintain oral nutrition were also excluded. Consequently, a total of 237 patients were included in this study. This study was approved by the Institutional Review Board (IRB) of Soonchunhyang University Cheonan Hospital (IRB approval number: 2024-05-045, IRB approval date: 26 June 2024). Written informed consent was obtained from all patients.

The ONS group received commercial products (Encover, 200 mL, containing 8.76 g protein and 200 kcal per serving, JW Pharmaceutical, Seoul, Korea) for nutritional support twice daily from the first postoperative day until hospital discharge, in addition to oral meals. The control group maintained oral meals without ONSs. The control group included some patients who underwent hip fracture surgery before the initiation of ONS administration in our institution, as well as those who refused to take ONSs. Surgeries were performed using routine methods of joint replacement under general or spinal anesthesia with a standardized protocol by a single anesthesiologist. All patients received standardized management of comorbidities according to hospital protocols before and after surgery. Blood transfusion was performed restrictively only if hemoglobin levels were below 8 g/dL or if symptomatic anemia was present [10]. If patients had poor oral intake and hypoalbuminemia (serum albumin level < 3 g/dL), 20% albumin was administered intravenously. Intravenous patient-controlled analgesia was applied to all patients postoperatively. Venous thromboembolism prophylaxis was administered according to domestic guidelines [11]. Postoperative recovery exercises were conducted according to standardized protocols of our institution for all patients. Patients were allowed to use a wheelchair from the first day after surgery. Standing exercises were started on the second day after surgery, progressing to gradual walking exercises based on the patient’s tolerance level. All patients were discharged from the hospital when they demonstrated systemic improvement approximately two weeks after surgery.

Primary outcomes were differences in serum protein and albumin levels between the two groups at two weeks post operation. Secondary outcomes were the degree of change in serum protein and albumin levels before and after surgery, the requirement of albumin replacement, the length of hospital stay after surgery, and the incidence of postoperative complications. Data collected as postoperative complications included urinary tract infection (UTI), paralytic ileus, pneumonia, and delirium.

Data were analyzed using SPSS 27.0 (IBM, Chicago, IL, USA). Descriptive statistics were employed to characterize patients. Continuous variables with normal distribution were presented as mean ± standard deviation (SD) and compared between groups using the Student’s *t*-test. Categorical variables were expressed as frequencies and percentages, and comparisons between groups were conducted using the chi-square or Fisher’s exact test as needed. Perioperative hematologic parameter changes and length of hospital stay were normally distributed and were compared between groups using the Student’s *t*-test. Differences in diagnosis of THA, past medical history, and postoperative complication rates between groups were analyzed using the chi-square test. A *p* < 0.05 was considered statistically significant.

## 3. Results

### 3.1. Patient Demographics

This study included a total of 237 patients, with 69 receiving postoperative ONSs and 168 not receiving ONSs. There were no significant differences in baseline characteristics such as age, sex, past medical history, or diagnosis of THA between the two groups (Table 1).

### 3.2. Outcomes

Comparisons of perioperative hematologic parameters between the two groups are detailed in Table 2.

**Figure 1:**
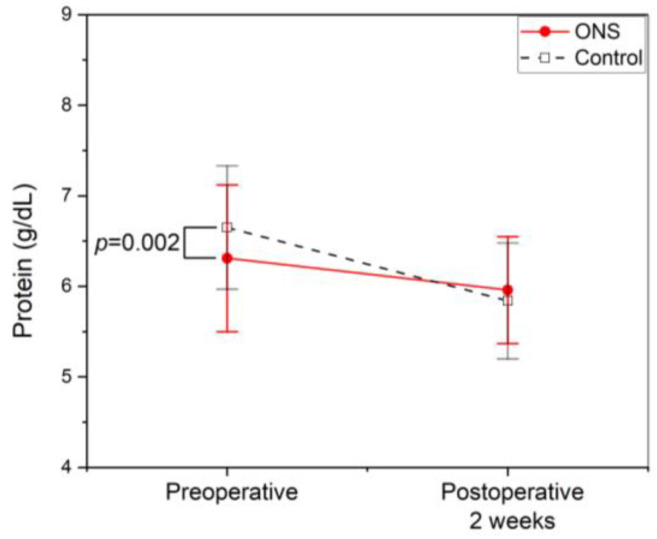
Profile of serum protein. Symbols represent means, and error bars show the standard deviation. Filled circles with a straight line represent the oral nutritional supplement (ONS) group, whereas open squares with a dotted line are controls. The mean preoperative serum protein level was significantly higher in the control group. However, it showed no significant difference at two weeks post operation between the two groups. The decline in serum protein was significantly less in the ONS group (*p* < 0.001).

While the mean preoperative serum protein was higher in the control group (6.65 ± 0.68 vs. 6.31 ± 0.81 g/dL, *p* = 0.002), the decline in serum protein level from pre-operation to two weeks after surgery was significantly less in the ONS group (0.35 ± 0.87 vs. 0.81 ± 0.61 g/dL, *p* < 0.001). As a result, there was no significant difference in mean serum protein at two weeks post operation (5.96 ± 0.59 Vs. 5.84 ± 0.64 g/dL, *p* = 0.210) (Figure 1).

**Figure 2:**
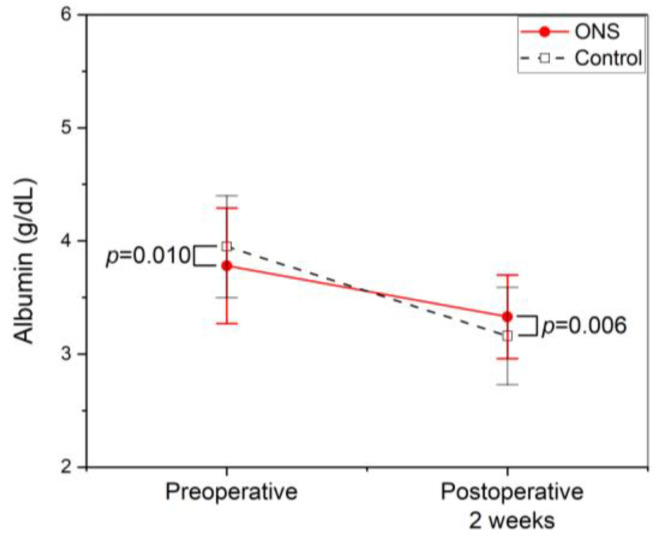
Profile of serum albumin. Symbols represent means, and error bars show the standard deviation. Filled circles with a straight line represent the oral nutritional supplement (ONS) group, whereas open squares with a dotted line represent controls. The mean serum albumin level was significantly higher in the control group preoperatively. However, at two weeks post operation, it was significantly higher in the ONS group. The decline in serum albumin was significantly less in the ONS group (*p* < 0.001).

Similarly, the mean preoperative serum albumin was higher in the control group (3.95 ± 0.45 vs. 3.78 ± 0.51 g/dL, *p* = 0.010), but the decline in serum albumin level was significantly less in the ONS group (0.45 ± 0.52 vs. 0.79 ± 0.39 g/dL, *p* < 0.001). The mean serum albumin at two weeks post operation was significantly higher in the ONS group (3.33 ± 0.37 vs. 3.16 ± 0.43 g/dL, *p* = 0.006) (Figure 2).

However, there was no significant difference in the albumin replacement during the hospitalization period between the two groups (0.74 ± 1.61 vs. 0.59 ± 1.07, *p* = 0.402).

The length of hospital stay after surgery was significantly shorter in the ONS group (15.64 ± 4.03 vs. 18.52 ± 8.33 days, *p* < 0.001). When comparing the incidence of postoperative complications between the two groups, postoperative delirium occurred significantly more frequently in the control group (46.43% vs. 27.54%, *p* = 0.007). There were no significant differences in the incidence of UTI, paralytic ileus, or pneumonia (Table 3).

## 4. Discussion

Efforts to diagnose and treat concurrent comorbidities such as osteoporosis, sarcopenia, and cachexia in geriatric patients admitted for acute care are a global concern. Malnutrition is not only more common than the aforementioned conditions in geriatric patients but also deemed the most detrimental comorbidity, serving as a significant risk factor for unfavorable postoperative outcomes [12,13,14]. Furthermore, malnutrition contributes to prolonged length of hospital stay and represents the comorbidity requiring the highest costs related to hospitalization [15]. Elderly patients admitted for hip fractures are particularly at a significantly high risk of malnutrition, with over half of them being diagnosed with this condition upon admission [1,16]. However, in real-world settings, efforts to correct the nutritional status of geriatric hip fracture patients are often not recognized as a priority treatment. They seem to be undervalued in terms of importance. Decreased anabolic response, exacerbation of catabolism due to acute fractures in addition to chronic underlying conditions, and underlying sarcopenia all can contribute to increased energy and protein requirements in elderly patients. While healthy geriatrics typically require around 25–30 kcal/kg of energy and 1 g/kg of protein per day, patients with hip fractures may require even higher amounts of energy and protein supply [17,18]. This study aimed to investigate whether providing a product containing 8.76 g of protein twice daily, without calculating weight-adjusted protein requirements, could improve the nutritional status of geriatric hip fracture patients and reduce postoperative complications. Through this, we sought to demonstrate whether minimal interventions, rather than specialized nutritional management, could yield beneficial effects.

We evaluated changes in serum protein and albumin levels to assess the effectiveness of ONSs. These parameters are widely utilized as indicators for assessing nutritional status due to their cost-effectiveness and ease of results interpretation. These values not only indicate protein status but also serve as predictors of functional prognosis in elderly patients. In a retrospective study including geriatric patients diagnosed with distal radius fracture, a decrease in serum albumin level was found to be significantly associated with an increased risk of subsequent falls [19]. Administering ONS appears to have an effect on reducing falls by improving hematological parameters associated with nutritional status [20]. The results of this study demonstrated that postoperative administration of ONSs could effectively improve nutritional status in geriatric hip fracture patients undergoing THA. ONSs are designed to provide a variety of nutrients to individuals with inadequate dietary intake. Regardless of the product’s form or dosage, hospitalized elderly patients are strongly recommended to receive ONSs. European guidelines strongly recommend postoperative ONS administration for geriatric hip fracture patients irrespective of their nutritional status to enhance dietary intake and reduce complications [3]. Interestingly, the Cochrane review and meta-analysis that formed the basis for this recommendation found that standard ONS supplementation generally could increase energy intake in most patients, although special ONSs with high protein content (>20% energy from protein) did not confer additional benefits in terms of complication rates or mortality [9]. Clinical nutrition guidelines in Asia also strongly recommend the use of ONSs to mitigate nutritional risks and reduce postoperative complications in patients with hip fractures [21]. Moreover, for patients with dementia, nutritional education alone may be insufficient, and administration of ONSs is advised to ensure adequate energy intake. Botella-Carretero et al. [22] performed a randomized clinical trial and found that postoperative ONS has no benefits for general postoperative nutritional status or length of hospital stay, although it could benefit patients with comorbidities and longer hospitalizations. Given that the serum albumin level has already decreased postoperatively, the authors suggest that continuous ONS administration from admission to hospital discharge could be more effective than postoperative administration alone [23]. However, our study results demonstrated the effectiveness of providing ONSs postoperatively in improving nutritional status.

Just because oral intake is possible, relying solely on meals provided may not adequately meet nutritional requirements. While individualized nutritional assessment and intervention are crucial, they may demand significant service requirements and increase healthcare cost burdens. As mentioned earlier, elderly patients with hip fractures are all at high risk of malnutrition. Therefore, it is advisable to supply high-quality multi-nutrients to all patients uniformly. In this regard, simply adding standardized ONSs to regular meals can be highly promising for improving postoperative nutritional status. Efforts to enhance nutritional status by providing multi-nutrient supplementation should be recognized as an essential step in treating hip fracture patients, given the difficulty in meeting daily nutritional requirements by regular meals alone.

In our study, postoperative ONSs also resulted in a shortened length of hospital stay. A retrospective study conducted using a healthcare database in the United States similarly found that postoperative ONS administration significantly reduced the length of hospitalization in malnourished patients with hip fracture [24]. The length of hospital stay for patients receiving ONSs from 1 day post operation was significantly shorter, with a mean of 5.8 days compared to 7.6 days for patients not receiving ONSs. That study found no association of earlier ONS administration with a reduction in infection rates, in-hospital mortality, or ICU admission rate.

In the present study, the ONS group experienced approximately 19% less postoperative delirium than the control group. Postoperative delirium is common, occurring in over half of geriatric patients with hip fracture [25]. Elderly patients diagnosed with preoperative malnutrition prior to hip fracture surgery experience nearly a three-fold increase in postoperative delirium compared to well-nourished patients [26]. Strategies for mitigating postoperative delirium following hip fracture surgery include fluid management, pain control, early mobilization, optimizing the bedside environment, and electrolyte correction, along with nutritional improvements. These interventions can effectively reduce the incidence and severity of postoperative delirium [27]. Therefore, a multidimensional, non-pharmacological approach to preventing delirium must include nutritional management.

In real-world practice, proactive assessment of nutritional status is lacking and provision of nutritional supplementation is inadequate. In the aforementioned retrospective study, the authors pointed out that among all hip fracture patients, only 1.6% received nutritional supplements [24]. Even among patients diagnosed with nutritional deficiencies, only 4.9% were prescribed early nutritional supplementation. Another study found that among 8713 malnourished adults, only 3.1% received ONSs [28]. Despite nutritional guidelines strongly recommending ONSs in elderly patients, the majority of patients still do not receive ONS prescriptions. In this regard, the meaning of this study lies in confirming the benefits of postoperative ONS administration for improving parameters of good clinical outcomes.

This study has several limitations. Firstly, relying solely on serum albumin and protein to assess nutritional status could be inaccurate, as they could be influenced by non-nutritional factors such as inflammation and aging [16]. Secondly, this study was a retrospective analysis of existing data. Conducting a prospective controlled study could enhance the reliability of the study results by minimizing potential biases and confounding variables. Third, this study was a single-center study with a relatively small number of patients included. This study did not assess the differences in outcomes based on the anesthesia method. Considering that regional anesthesia may reduce the length of hospital stay compared to general anesthesia in hip fracture surgery, differences in anesthesia methods could be a possible confounding factor. Further research on this aspect is warranted [29]. Additionally, since this study only included patients who continued ONSs until discharge, factors for discontinuation of ONSs and compliance were not assessed. Furthermore, patients admitted to the ICU postoperatively and those transferred to other departments were excluded from this study. Thus, we could not evaluate the effect of ONSs on severe complications requiring ICU admission or transfer. As mentioned earlier, we did not control for confounding variables in patients who required additional treatments; thus, they could not be included in the data. Therefore, a prospective study that controls for these variables is being prepared and is expected to yield more reliable results and provide further evidence supporting the benefits of ONSs in geriatric patients with hip fractures.

In geriatric patients with hip fractures who are susceptible to malnutrition, postoperative ONSs are strongly recommended. However, it is not often prescribed in clinical settings. The results of this study suggest that providing ONSs alongside meals can improve postoperative nutritional status and reduce the incidence of postoperative delirium. This demonstrates that even minimal nutritional interventions can yield beneficial effects without the need for specialized nutritional assessments. These effects ultimately can shorten the recovery process after surgery and reduce hospitalization duration. Therefore, the administration of ONSs to elderly patients undergoing hip fracture surgery should be considered an essential component of postoperative recovery strategies.

## 5. Conclusions

Postoperative ONSs in geriatric hip fracture patients can improve nutritional status, reduce the length of hospital stay, and decrease the incidence of postoperative delirium.

## Figures and Tables

**Table 1:** Patient demographics.

	ONSs (n = 69)	Controls (n = 168)	*p*-Value
**Age (years)**	80.93 ± 6.83	80.89 ± 8.32	0.971
**Sex (%)**			0.063
Male	9 (13.04)	40 (23.81)	
Female	60 (86.96)	128 (76.19)	
**Diagnosis of THA (%)**			
Femur neck fracture	57 (82.61)	147 (87.50)	0.323
Intertrochanteric fracture	11 (15.94)	20 (11.90)	0.402
Internal fixation failure	1 (1.45)	1 (0.60)	0.498
**Past history (%)**			
Hypertension	52 (75.36)	119 (70.83)	0.480
Diabetes	23 (33.33)	59 (35.12)	0.793
Chronic kidney disease	6 (8.70)	26 (15.48)	0.165
Liver disease	2 (2.90)	12 (7.14)	0.362
Heart disease	20 (28.99)	48 (28.57)	0.949
Cerebrovascular disease	10 (14.49)	40 (23.81)	0.110
Malignancy	11 (15.94)	20 (11.90)	0.402

ONSs, oral nutritional supplements; THA, total hip arthroplasty.

**Table 2:** Comparisons of perioperative hematologic parameters.

	ONS	Controls	*p*-Value
**Protein (g/dL)**			
Preoperative	6.31 ± 0.81	6.65 ± 0.68	0.002
Two weeks post operation	5.96 ± 0.59	5.84 ± 0.64	0.210
Change in protein	−0.35 ± 0.87	−0.81 ± 0.61	<0.001
**Albumin (g/dL)**			
Preoperative	3.78 ± 0.51	3.95 ± 0.45	0.010
Two weeks post operation	3.33 ± 0.37	3.16 ± 0.43	0.006
Change in albumin	−0.45 ± 0.52	−0.79 ± 0.39	<0.001
**Albumin replacement**	0.74 ± 1.61	0.59 ± 1.07	0.402

ONS, oral nutritional supplements.

**Table 3:** Comparisons of the incidence of postoperative complications and length of hospital stay.

	ONS	Controls	*p*-Value
**Complications (%)**			
Urinary tract infection	10 (14.49)	31 (18.45)	0.464
Paralytic ileus	4 (5.80)	11 (6.55)	>0.999
Pneumonia	6 (8.70)	21 (12.50)	0.402
Delirium	19 (27.54)	78 (46.43)	0.007
**Hospitalization period after surgery (days)**	15.64 ± 4.03	18.52 ± 8.33	<0.001

ONS, oral nutritional supplements.

## Data Availability

The original contributions presented in the study are included in the article.

## References

[1] Bell J., Bauer J., Capra S., Pulle C.R. (2013). Barriers to nutritional intake in patients with acute hip fracture: Time to treat malnutrition as a disease and food as a medicine?. Can. J. Physiol. Pharmacol..

[2] Malafarina V., Reginster J.Y., Cabrerizo S., Bruyere O., Kanis J.A., Martinez J.A., Zulet M.A. (2018). Nutritional Status and Nutritional Treatment Are Related to Outcomes and Mortality in Older Adults with Hip Fracture. Nutrients.

[3] Volkert D., Beck A.M., Cederholm T., Cruz-Jentoft A., Goisser S., Hooper L., Kiesswetter E., Maggio M., Raynaud-Simon A., Sieber C.C. (2019). ESPEN guideline on clinical nutrition and hydration in geriatrics. Clin. Nutr..

[4] Rempel A.N., Rigassio Radler D.L., Zelig R.S. (2023). Effects of the use of oral nutrition supplements on clinical outcomes among patients who have undergone surgery for hip fracture: A literature review. Nutr. Clin. Pract. Off. Publ. Am. Soc. Parenter. Enter. Nutr..

[5] Delmi M., Rapin C.H., Bengoa J.M., Delmas P.D., Vasey H., Bonjour J.P. (1990). Dietary supplementation in elderly patients with fractured neck of the femur. Lancet.

[6] Tkatch L., Rapin C.H., Rizzoli R., Slosman D., Nydegger V., Vasey H., Bonjour J.P. (1992). Benefits of oral protein supplementation in elderly patients with fracture of the proximal femur. J. Am. Coll. Nutr..

[7] Milne A.C., Potter J., Avenell A. (2002). Protein and energy supplementation in elderly people at risk from malnutrition. Cochrane Database Syst. Rev..

[8] Hubbard G.P., Elia M., Holdoway A., Stratton R.J. (2012). A systematic review of compliance to oral nutritional supplements. Clin. Nutr..

[9] Avenell A., Smith T.O., Curtain J.P., Mak J.C., Myint P.K. (2016). Nutritional supplementation for hip fracture aftercare in older people. Cochrane Database Syst. Rev..

[10] Carson J.L., Terrin M.L., Noveck H., Sanders D.W., Chaitman B.R., Rhoads G.G., Nemo G., Dragert K., Beaupre L., Hildebrand K. (2011). Liberal or restrictive transfusion in high-risk patients after hip surgery. N. Engl. J. Med..

[11] Park Y.-S. (2011). Guideline for the prophylaxis of venous thromboembolism in hip surgery patients. J. Korean Orthop. Assoc..

[12] Barker L.A., Gout B.S., Crowe T.C. (2011). Hospital malnutrition: Prevalence, identification and impact on patients and the healthcare system. Int. J. Environ. Res. Public Health.

[13] DiMaria-Ghalili R.A. (2002). Changes in nutritional status and postoperative outcomes in elderly CABG patients. Biol. Res. Nurs..

[14] Baldwin C., Parsons T.J. (2004). Dietary advice and nutritional supplements in the management of illness-related malnutrition: Systematic review. Clin. Nutr..

[15] Nikkel L.E., Fox E.J., Black K.P., Davis C., Andersen L., Hollenbeak C.S. (2012). Impact of comorbidities on hospitalization costs following hip fracture. J. Bone Jt. Surg. Am. Vol..

[16] Bell J.J., Bauer J.D., Capra S., Pulle R.C. (2014). Concurrent and predictive evaluation of malnutrition diagnostic measures in hip fracture inpatients: A diagnostic accuracy study. Eur. J. Clin. Nutr..

[17] Gaillard C., Alix E., Sallé A., Berrut G., Ritz P. (2007). Energy requirements in frail elderly people: A review of the literature. Clin. Nutr..

[18] Bauer J., Biolo G., Cederholm T., Cesari M., Cruz-Jentoft A.J., Morley J.E., Phillips S., Sieber C., Stehle P., Teta D. (2013). Evidence-based recommendations for optimal dietary protein intake in older people: A position paper from the PROT-AGE Study Group. J. Am. Med. Dir. Assoc..

[19] Nagai T., Tanimoto K., Tomizuka Y., Uei H., Nagaoka M. (2020). Nutrition status and functional prognosis among elderly patients with distal radius fracture: A retrospective cohort study. J. Orthop. Surg. Res..

[20] Gray-Donald K., Payette H., Boutier V. (1995). Randomized clinical trial of nutritional supplementation shows little effect on functional status among free-living frail elderly. J. Nutr..

[21] Wei J., Chen W., Zhu M., Cao W., Wang X., Shi H., Dong B., Sun J., Chen H., Zhou Y. (2015). Guidelines for parenteral and enteral nutrition support in geriatric patients in China. Asia Pac. J. Clin. Nutr..

[22] Botella-Carretero J.I., Iglesias B., Balsa J.A., Zamarrón I., Arrieta F., Vázquez C. (2008). Effects of oral nutritional supplements in normally nourished or mildly undernourished geriatric patients after surgery for hip fracture: A randomized clinical trial. JPEN. J. Parenter. Enter. Nutr..

[23] Botella-Carretero J.I., Iglesias B., Balsa J.A., Arrieta F., Zamarrón I., Vázquez C. (2010). Perioperative oral nutritional supplements in normally or mildly undernourished geriatric patients submitted to surgery for hip fracture: A randomized clinical trial. Clin. Nutr..

[24] Williams D.G.A., Ohnuma T., Haines K.L., Krishnamoorthy V., Raghunathan K., Sulo S., Cassady B.A., Hegazi R., Wischmeyer P.E. (2021). Association between early postoperative nutritional supplement utilisation and length of stay in malnourished hip fracture patients. Br. J. Anaesth..

[25] Bruce A.J., Ritchie C.W., Blizard R., Lai R., Raven P. (2007). The incidence of delirium associated with orthopedic surgery: A meta-analytic review. Int. Psychogeriatr..

[26] Mazzola P., Ward L., Zazzetta S., Broggini V., Anzuini A., Valcarcel B., Brathwaite J.S., Pasinetti G.M., Bellelli G., Annoni G. (2017). Association Between Preoperative Malnutrition and Postoperative Delirium After Hip Fracture Surgery in Older Adults. J. Am. Geriatr. Soc..

[27] Marcantonio E.R., Flacker J.M., Wright R.J., Resnick N.M. (2001). Reducing delirium after hip fracture: A randomized trial. J. Am. Geriatr. Soc..

[28] Mullin G.E., Fan L., Sulo S., Partridge J. (2019). The Association between Oral Nutritional Supplements and 30-Day Hospital Readmissions of Malnourished Patients at a US Academic Medical Center. J. Acad. Nutr. Diet..

[29] Neuman M.D., Rosenbaum P.R., Ludwig J.M., Zubizarreta J.R., Silber J.H. (2014). Anesthesia technique, mortality, and length of stay after hip fracture surgery. JAMA.

